# Treatment with pemafibrate ameliorates fatty liver index and atherogenic lipid profiles in Japanese patients with type 2 diabetes mellitus

**DOI:** 10.3389/fendo.2025.1496671

**Published:** 2025-07-17

**Authors:** Toru Suzuki, Tatsuya Sato, Marenao Tanaka, Kei Nakata, Keisuke Endo, Hiroki Aida, Wataru Kawaharata, Itaru Hosaka, Araya Umetsu, Toshifumi Ogawa, Yukinori Akiyama, Masato Furuhashi

**Affiliations:** ^1^ Department of Cardiovascular, Renal and Metabolic Medicine, Sapporo Medical University School of Medicine, Sapporo, Japan; ^2^ Natori Toru Internal Medicine and Diabetes Clinic, Natori, Japan; ^3^ Department of Cellular Physiology and Signal Transduction, Sapporo Medical University School of Medicine, Sapporo, Japan; ^4^ Tanaka Medical Clinic, Yoichi, Japan; ^5^ Department of Public Health, Sapporo Medical University School of Medicine, Sapporo, Japan; ^6^ Department of Cardiovascular Surgery, Sapporo Medical University School of Medicine, Sapporo, Japan; ^7^ Department of Ophthalmology, Sapporo Medical University School of Medicine, Sapporo, Japan; ^8^ Department of Neurosurgery, Sapporo Medical University School of Medicine, Sapporo, Japan

**Keywords:** pemafibrate, fatty liver index, metabolic dysfunction-associated steatotic liver disease, type 2 diabetes, atherogenic lipid profiles

## Abstract

**Background:**

Pemafibrate, a selective peroxisome proliferator-activated receptor α modulator, ameliorates hypertriglyceridemia. We investigated the effects of pemafibrate on steatotic liver disease (SLD) in relation to various atherogenic lipid profiles.

**Methods:**

Thirty-nine Japanese patients with both type 2 diabetes mellitus (T2DM) and hypertriglyceridemia (men/women: 24/15, mean age: 58.2 years, median duration of diabetes: 5.0 years) were treated with 0.2 mg/day of pemafibrate for 12 months (M). SLD was estimated by fatty liver index (FLI), which is calculated by using waist circumference, body mass index and levels of triglycerides and γ-glutamyl transpeptidase.

**Results:**

Treatment with pemafibrate significantly increased mean levels of high-density lipoprotein cholesterol (HDL-C) (baseline/3M/6M/12M: 46/55/55/54 mg/dL) and decreased median levels of triglycerides (baseline/3M/6M/12M: 211/112/99/98 mg/dL), non-HDL-C (146/128/125/121 mg/dL), small dense low-density lipoprotein cholesterol (45/33/30/30 mg/dL) and remnant-like particle cholesterol (8.1/2.6/2.3/2.4 mg/dL). There was no significant change in hemoglobin A1c level over time. FLI (mean ± standard deviation: 68.1 ± 21.9 *vs*. 39.6 ± 25.0, P < 0.001), but not FIB-4 index as a marker of hepatic fibrosis (median [interquartile range]: 1.04 [0.78-1.39] *vs*. 1.01 [0.68-1.36], P = 0.909), was significantly decreased by treatment with pemafibrate for 12M, and the proportion of patients with metabolic dysfunction-associated SLD (MASLD) was significantly decreased from 92.3% (baseline) to 61.5% (12M).

**Conclusions:**

Pemafibrate ameliorates MASLD estimated by FLI in addition to various atherogenic lipid profiles in Japanese hypertriglyceridemia patients with T2DM in the past mean 5 years. An early intervention with pemafibrate might contribute to prevention of the development of MASLD and atherosclerotic cardiovascular disease.

## Introduction

Atherosclerotic cardiovascular disease (ASCVD) is one of the major health problems worldwide (1). Low-density lipoprotein (LDL) cholesterol (LDL-C) has been shown as a therapeutic target for ASCVD (2). Various lipid abnormalities have also been reported to be targets as residual risks for ASCVD, and the candidates include small dense LDL (sdLDL) cholesterol (sdLDL-C) and remnant-like particle cholesterol (RLP-C) (3). sdLDL contains smaller amounts of apoprotein (Apo) B (ApoB) and ApoE, ligands for LDL receptors in hepatocytes, resulting in a longer stay of sdLDL-C than that of LDL-C in the blood (4). Furthermore, sdLDL infiltrates into the outer vascular space and can be easily oxidized, resulting in the progression of atherosclerosis (4). On the other hand, RLP-C is determined by intermediate metabolites resulting from the breakdown of lipoproteins such as very low-density lipoprotein (VLDL) and intermediate-density lipoprotein (IDL) (5) and can induce ASCVD by being easily taken up by phagocytic cells and by having aggregating effects on platelets (6).

Nonalcoholic fatty liver disease (NAFLD) has been reported to be an upstream risk factor for atherosclerosis (7–9). New nomenclature of steatotic liver disease (SLD) including metabolic dysfunction-associated SLD (MASLD) has recently been proposed (10). Individuals with SLD complicated with diabetes mellitus (DM) are defined as patients who have MASLD or MASLD and increased alcohol intake (MetALD) (10). Although MASLD can be a potent risk for ASCVD (11) as well as its related diseases (12, 13), there are no effective and specific agents for treatment of MASLD. Fatty liver index (FLI) (14), which is calculated by using waist circumference (WC), body mass index (BMI) and levels of triglycerides (TG) and γ-glutamyl transpeptidase (γGT), has been established as a useful biomarker for detection of SLD (12, 15) as well as for risk estimation for the development of hypertension (16), DM (17), chronic kidney disease (18, 19) and ischemic heart disease (20). Thus, SLD is an upstream factor in cardiorenal metabolic diseases and may be a promising therapeutic target for ASCVD.

Pemafibrate, a selective peroxisome proliferator-activated receptor α (PPARα) modulator (SPARMα), has recently been developed for treatment of hypertriglyceridemia as a residual risk for ASCVD (21) as well as for improvement of NAFLD (22–25). The PROMINENT trial using patients with type 2 DM (T2DM) and hypertriglyceridemia revealed that treatment with pemafibrate did not reduce cardiovascular events over a median observation period of 3.4 years (22). In that trial, the recruited patients who had been treated with statins (96%, high-intensity dose: 69%) had mean levels of LDL-C as low as 79 mg/dL at baseline, and levels of non-HDL-C were not significantly changed by treatment with pemafibrate (22, 26). In a pooled analysis of the PROMINENT trial (27), levels of sdLDL-C estimated by the Sampson equation (28, 29), which has been validated by several cohorts (30–33), were not reduced by treatment with pemafibrate regardless of a reduction in TG level, which might be one possible reason why cardiovascular events were not reduced in the PROMINENT trial (22). In addition, the possibility that the relatively long duration of DM [≥10 years: 46.4% (22)] in the recruited patients affected the outcome due to the presence of already advanced latent atherosclerosis cannot be ruled out.

To address our hypothesis, we prospectively investigated the effects of pemafibrate on SLD assessed by FLI in relation to various atherogenic lipid profiles in Japanese patients with hypertriglyceridemia and T2DM who have a relatively short duration of DM.

## Methods

This study was a prospective single-center observational study conducted in Japan. The study conformed to the principles outlined in the Declaration of Helsinki and was approved by the human ethics committee of Natori Toru Internal Medicine and Diabetes Clinic (approval number: CR2022-01). Written informed consent was obtained from all of the subjects.

### Study patients

Study patients were enrolled from outpatients attending Natori Toru Internal Medicine and Diabetes Clinic (Natori, Japan) who agreed to participate in the study. The inclusion criteria were as follows: 1) diagnosis of T2DM; 2) fasting serum TG > 150 mg/dL; 3) serum creatinine < 2.5 mg/dL; 4) age ≥ 18 years old; and 5) Japanese race. The exclusion criteria included any of the following: 1) patients treated with drugs for hypertriglyceridemia including fibrates, omega-3 polyunsaturated fatty acids, eicosatetraenoic acid and docosahexaenoic acid; 2) patients treated with steroids; 3) presence of diseases that can affect the serum level of TG including nephrotic syndrome, Cushing’s syndrome, inadequately controlled hypothyroidism, primary biliary cholangitis and obstructive jaundice; 4) unstable condition with progressive multiple organ damage; and 5) patients who were pregnant or potentially childbearing. According to the standard drug information, the recruited patients were treated with pemafibrate at a dose of 0.2 mg/day (0.1 mg twice daily) for 12 months.

### Clinical examinations

The participants were examined every month to check their health status and the presence of any side effects of pemafibrate including any musculoskeletal, renal and hepatic events. Detailed laboratory parameters were measured before the start of treatment with pemafibrate and at 3 months, 6 months and 12 months after the start of treatment. Blood samples were collected in the morning in an overnight fasting condition.

Body weight was measured to the nearest 0.1 kg using a digital scale, and height was measured to the nearest 0.1 cm. BMI was calculated as body weight (kg)/(height [m])^2^. WC was measured at the umbilical level in the late phase of expiration to the nearest 0.1 cm. Systolic blood pressure, diastolic blood pressure and pulse rate were measured by using a fully automatic measuring device (HBP-9020, Omron, Japan). A self-administered questionnaire survey was performed to obtain information on current smoking habit and alcohol drinking habit (≥ 1 time/week).

### Measurements

Levels of total cholesterol (TC), high-density lipoprotein cholesterol (HDL-C), TG, and RLP-C were measured by enzymatic assays. sdLDL-C concentration was directly measured by using a homogenous assay (sdLDL-EX SEIKEN; Denka Co., Tokyo, Japan) (34, 35). Lipoprotein (a) (Lp(a)) and apolipoproteins including ApoA1, ApoA2, ApoB, ApoC2, ApoC3 and ApoE were measured by turbidimetric immunoassay methods (BML, Inc., Tokyo, Japan). Lipoprotein fractions were measured by using a high-performance liquid chromatography (HPLC) method (BML, Inc., Tokyo, Japan) (36, 37).

LDL-C was calculated by using the Friedewald formula (38): TC − HDL-C − TG/5. Non-HDL-C was calculated by subtracting HDL-C from TC. TG-rich lipoprotein cholesterol (TRL-C), which is the same as remnant cholesterol reported in previous studies (39–41), was calculated by subtracting HDL-C and LDL-C from TC. Estimated glomerular filtration rate (eGFR) was calculated by the following equation for Japanese people (42): eGFR (mL/min/1.73m^2^) = 194 × serum creatinine^-1.094^ × age^-0.287^ × 0.739 (if female).

### FLI and MASLD

Fatty liver index (FLI) was calculated by the following formula (14): [e^(0.953 × ln TG + 0.139 × BMI + 0.718 × ln(γGT) + 0.053 × WC – 15.745)^]/[1 + e ^(0.953 × ln TG + 0.139 × BMI + 0.718 × ln(γGT) + 0.053 × WC – 15.745)^] × 100. Although the cutoff value for SLD was originally reported as FLI ≥ 60 in Italian subjects (14), FLI ≥ 35 for men and FLI ≥ 16 for women were used for the definition of SLD in the present study as previously reported in Japanese subjects (15). FIB-4 index, a marker of hepatic fibrosis, was also calculated by the following formula (43): age (years) × aspartate aminotransferase (AST; IU/L))/(platelet count [10^9^/L] × alanine aminotransferase [ALT; IU/L]^1/2^).

MASLD was diagnosed by the absence of other discernible causes for hepatic steatosis and the presence of SLD with at least one of five cardiometabolic risk factors assessed by BMI, glucose management, blood pressure and levels of TG and HDL-C (10). The five cardiometabolic criteria include 1) BMI ≥ 23 or WC > 90/80 cm in Asian men and women; 2) fasting glucose ≥ 100 mg/dL, 2-h post-load glucose levels ≥ 140 mg/dL (no measurement in the present study), hemoglobin A1c (HbA1c) ≥ 5.7%, type 2 diabetes mellitus, or treatment for type 2 diabetes mellitus; 3) blood pressure ≥ 130/85 mmHg or specific antihypertensive drug treatment; 4) plasma TG ≥ 150 mg/dL or lipid-lowering treatment; and 5) plasma HDL cholesterol ≤ 40 mg/dL for men and ≤ 50 mg/dL for women or lipid-lowering treatment. MetALD was diagnosed by the presence of MASLD and average alcohol intake of 140–350 g/week [20–50 g/day] for women and 210–420 g/week [30–60 g/day] for men.

### Statistical analysis

Numeric variables are expressed as means ± standard deviation (SD) for parameters with normal distributions and as medians [interquartile ranges] for parameters with skewed distributions. The distribution of each parameter was tested for its normality using the Shapiro-Wilk W test. Comparisons between two groups for parametric and nonparametric factors were performed by using Student’s t-test and the Mann-Whitney U test, respectively. Paired categorical indices were statistically compared by McNemar’s test. For comparison of two variables paired with time series correspondence, Wilcoxon single rank test was used. For comparison of three and more variables paired with time series correspondence, the Friedman test with Dunn’s *post-hoc* test was used. A p value of less than 0.05 was considered statistically significant. All data were analyzed by using EZR (44) and GraphPad Prism version 9.5.

## Results

### Characteristics of the study patients

Characteristics of the 39 recruited patients (men/women: 24/15) at baseline are shown in **Table 1**. The mean age of the patients was 58.2 ± 13.1 years, and 61.5% of the patients were men. The mean duration of DM was 5.0 [1.5-12.0] years. SLD was present in 32 patients (92.3%, men/women: 24/12) at determined by FLI ≥ 35 for men and FLI ≥ 16 for women, which were the cutoff values of SLD previously reported in Japanese subjects (15). Only 4 male patients had an alcohol drinking habit, and all of those patients had less than 30 g/day of alcohol equivalent. Subsequent interviews and clinical examinations revealed no evidence of alternative etiologies of SLD. Therefore, all of the patients with T2DM who had SLD were diagnosed as having MASLD but not MetALD. Only 1 female patient had a past history of ASCVD. Statins were used by 41.0% of patients (men/women: 9/7), and only 5.1% of those patients (men/women: 2/0) had been using ezetimibe.

**Table 1:** Characteristics of the recruited patients at baseline.

	All (n = 39)	Men (n = 24)	Women (n = 15)	*p*
Age (years)	58.2 ± 13.1	57.2 ± 14.5	59.7 ± 10.8	0.565
Body weight (kg)	74.9 ± 13.9	77.5 ± 14.4	70.9 ± 12.5	0.158
Waist circumference (cm)	95.3 ± 11.1	96.5 ± 10.1	93.4 ± 12.6	0.428
Body mass index (kg/m^2^)	27.5 ± 4.0	27.2 ± 3.9	27.9 ± 4.3	0.573
Systolic BP (mmHg)	131 [120-138]	132 [119-137]	131 [129-152]	0.312
Diastolic BP (mmHg)	75 [68-86]	77 [68-86]	75 [70-86]	0.729
Pulse rate (bpm)	83 [72-91]	84 [71-90]	80 [73-92]	0.942
Current smoking habit	9 (23.1)	9 (37.5)	0 (0.0)	0.021
Alcohol drinking habit	4 (10.3)	4 (16.7)	0 (0.0)	0.260
Duration of diabetes (years)	5.0 [1.5-12.0]	7.0 [2.8-13.5]	5.0 [1.0-9.0]	0.205
Diabetic microangiopathy				
Retinopathy	23 (59.0)	13 (54.2)	10 (66.7)	0.662
Nephropathy	14 (35.9)	8 (33.3)	6 (40.0)	0.937
Comorbidity				
MASLD	36 (92.3)	24 (61.5)	12 (30.8)	0.404
MetALD	0 (0)	0 (0)	0 (0)	–
Hypertension	29 (74.4)	16 (66.7)	13 (86.7)	0.31
ASCVD	1 (2.6)	0 (0.0)	1 (6.7)	0.81
Anti-lipidemic drugs				
Statin	16 (41.0)	9 (37.5)	7 (46.7)	0.817
Ezetimibe	2 (5.1)	2 (8.3)	0 (0.0)	0.688
Anti-diabetic drugs				
Biguanide	27 (69.2)	16 (66.7)	11 (73.3)	0.934
Thiazolidinedione	1 (2.6)	0 (0.0)	1 (6.7)	0.810
DPP-4 inhibitor	17 (43.6)	10 (41.7)	7 (46.7)	1.000
SGLT-2 inhibitor	19 (48.7)	15 (62.5)	4 (26.7)	0.065
Imeglimin	2 (5.1)	1 (4.2)	1 (6.7)	1.000
GLP-1 receptor agonist	18 (46.2)	11 (45.8)	7 (46.7)	1.000
Insulin	6 (15.4)	5 (20.8)	1 (6.7)	0.461

Variables are expressed as number (%), means ± SD or medians [interquartile ranges].

ASCVD, atherosclerotic cardiovascular disease; BP, blood pressure; DPP-4, dipeptidyl peptidase-4; GLP-1, glucagon-like peptide-1; MASLD, metabolic dysfunction-associated steatotic liver disease; MetALD, MASLD and increased alcohol intake; SGLT2, sodium-glucose cotransporter 2.

Biochemical data at baseline are shown in **Table 2**. Levels of fasting glucose and HbA1c were 117 [107-140] mg/dL and 6.8 [6.4-7.4]%, respectively. Levels of TG and LDL-C were 211 [183-262] mg/dL and 108 [84-127] mg/dL, respectively.

**Table 2:** Biochemical data at baseline.

	All (n = 39)	Men (n = 24)	Women (n = 15)	*p*
Biochemical data				
Fasting glucose (mg/dL)	117 [107-140]	119 [109-139]	117 [105-140]	0.862
Hemoglobin A1c (%)	6.8 [6.4-7.4]	6.9 [6.4-7.4]	6.8 [6.4-7.3]	0.783
Creatinine (mg/dL)	0.68 [0.57-0.79]	0.78 [0.63-0.91]	0.58 [0.49-0.74]	0.011
eGFR (mL/min/1.73m^2^)	83.7 ± 24.0	88.3 ± 25.7	84.4 ± 22.0	0.965
CK (IU/L)	93 [71-135]	98 [74-164]	90 [58-126]	0.156
Liver-related variables				
AST (IU/L)	24 [21-35]	24 [21-33]	23 [19-23]	0.954
ALT (IU/L)	29 [22-49]	28 [22-50]	34 [21-48]	0.817
γGT (IU/L)	35 [27-52]	42 [29-67]	28 [24-40]	0.055
ALP (IU/L)	75 [65-92]	74 [62-79]	78 [71-104]	0.236
FLI	67.8 ± 21.9	68.4 ± 20.8	66.7 ± 24.5	0.831
FIB-4	1.04 [0.78-1.39]	1.00 [0.80-1.45]	1.09 [0.75-1.23]	0.870
Lipid-related variables				
TG (mg/dL)	211 [183-262]	196 [179-231]	234 [199-285]	0.103
TC (mg/dL)	194 [179-228]	184 [150-205]	222 [193-256]	0.013
HDL-C (mg/dL)	46 ± 10	44 ± 10	50 ± 7	0.091
LDL-C (mg/dL)	108 [84-127]	98 [78-114]	127 [102-155]	0.012
non-HDL-C (mg/dL)	146 [130-172]	138 [116-155]	167 [144-203]	0.018
sdLDL-C (mg/dL)	45.4 [37.3-65.0]	41.9 [37.1-53.9]	60.0 [40.8-86.7]	0.051
sdLDL-C/LDL-C	0.47 [0.40-0.57]	0.43 [0.39-0.48]	0.54 [0.42-0.59]	0.102
RLP-C (mg/dL)	8.1 [6.2-10.1]	7.5 [5.8-9.4]	8.5 [7.9-12.8]	0.088
TRL-C (mg/dL)	56 [50-68]	52 [46-62]	60 [52-78]	0.083
Lp(a) (mg/dL)	3.5 [3.0-8.3]	3.0 [3.0-5.1]	8.0 [4.3-12.9]	0.001
Lipoprotein fraction				
VLDL-C (mg/dL)	41 [33-49]	35 [32-45]	37 [32-46]	0.119
IDL-C (mg/dL)	12 [10-15]	12 [9-14]	13 [12-17]	0.050
HDL-C (mg/dL)	42 ± 10	40 ± 10	45 ± 8	0.091
LDL-C (mg/dL)	94 [73-116]	90 [67-109]	99 [86-135]	0.163
Apolipoprotein fractions				
ApoA1 (mg/dL)	142.4 ± 22.4	136.3 ± 24.0	152.1 ± 15.8	0.030
ApoA2 (mg/dL)	32.3 ± 4.9	31.4 ± 4.9	33.9 ± 4.7	0.113
ApoB (mg/dL)	98.0 [86.0-115.0]	92.5 [78.3-105.3]	115.0 [94.0-136.0]	0.019
ApoC2 (mg/dL)	6.0 [5.2-6.9]	5.5 [4.8-6.3]	6.6 [5.7-7.5]	0.046
ApoC3 (mg/dL)	13.8 [12.2-16.9]	13.2 [11.9-16.0]	16.1 [12.3-18.4]	0.209
ApoC3/ApoC2	2.4 [2.0-2.7]	2.4 [2.1-2.7]	2.3 [1.9-2.7]	0.212
ApoE (mg/dL)	4.7 [4.0-6.0]	4.3 [3.6-5.0]	5.5 [4.7-6.6]	0.337

Variables are expressed as number (%), means ± SD or medians [interquartile ranges].

ALP, alkaline phosphatase; ALT, alanine transaminase; Apo, apolipoprotein; AST, aspartate transaminase; CK, creatine kinase; eGFR, estimated glomerular filtration rate; FIB-4, fibrosis-4; FLI, fatty liver index; γGT, γ-glutamyl transpeptidase; HDL-C, high-density lipoprotein cholesterol; IDL-C, intermediate density lipoprotein cholesterol; Lp(a), lipoprotein (a); LDL-C, low-density lipoprotein cholesterol; RLP-C, remnant-like particle cholesterol; sdLDL-C, small dense low-density lipoprotein cholesterol; TC, total cholesterol; TG, triglycerides; TRL-C, TG-rich lipoprotein cholesterol; VLDL-C, very low-density lipoproteins cholesterol.

### Changes in DM-related markers and side effects after the start of treatment with pemafibrate

Chronological changes in parameters after the start of treatment with pemafibrate are shown in **Table 3**. There was no significant change in HbA1c level after the start of treatment with pemafibrate, though fasting glucose level modestly, but significantly, decreased over time after the start of treatment with pemafibrate. Levels of creatinine and eGFR slightly, but significantly, decreased over time after the start of treatment with pemafibrate. Serum levels of creatine kinase, which is used to estimate the development of rhabdomyolysis, did not increase over time.

**Table 3:** Time course of physical and metabolic parameters.

	Baseline	3 months	6 months	12 months	*p*
Systolic BP (mmHg)	131 [120-138]	138 [127-148]	135 [128-149]	129 [116-137]	< 0.001
Diastolic BP (mmHg)	75 [68-86]	82 [75-86]	80 [70-88]	75 [69-82]	0.059
Pulse rate (bpm)	83 [72-91]	81 [75-88]	77 [70-85]	77 [72-88]	0.023
Biochemical data					
Fasting glucose (mg/dL)	117 [108-140]	111 [97-134]	106 [97-118]	100 [90-113]	0.003
Hemoglobin A1c (%)	6.9 [6.4-7.4]	6.7 [6.3-7.2]	6.6 [6.4-7.0]	6.6 [6.3-6.8]	0.810
Creatinine (mg/dL)	0.68 [0.57-0.79]	0.72 [0.59-0.96]	0.69 [0.59-1.00]	0.74 [0.60-1.04]	< 0.001
eGFR (mL/min/1.73m^2^)	83.7 ± 24.0	78.2 ± 25.3	78.8 ± 25.3	76.8 ± 23.2	< 0.001
CK (IU/L)	93 [71-135]	111 [71-158]	99 [66-161]	108 [75-145]	0.853
Lipid-related variables					
TG (mg/dL)	211 [183-262]	112 [79-139]	99 [83-120]	98 [76-141]	< 0.001
TC (mg/dL)	194 [179-228]	185 [167-201]	181 [163-197]	179 [160-193]	0.002
HDL-C (mg/dL)	46 ± 10	54 ± 13	55 ± 12	54 ± 13	< 0.001
LDL-C (mg/dL)	108 [84-127]	104 [95-119]	101 [90-121]	100 [87-109]	0.114
non-HDL-C (mg/dL)	146 [130-172]	128 [116-146]	125 [108-145]	121 [107-140]	< 0.001
sdLDL-C (mg/dL)	45.4 [37.3-65.0]	33.3 [25.3-40.5]	30.0 [22.9-37.0]	30.3 [22.6-38.3]	< 0.001
sdLDL-C/LDL-C	0.47 [0.40-0.57]	0.30 [0.24-0.36]	0.28 [0.23-0.36]	0.29 [0.25-0.35]	< 0.001
RLP-C (mg/dL)	8.1 [6.2-10.1]	2.6 [1.8-4.1]	2.3 [1.7-3.5]	2.4 [1.7-3.3]	< 0.001
TRL-C (mg/dL)	56 [55-68]	35 [25-44]	32 [22-36]	30 [24-39]	< 0.001
Lp(a) (mg/dL)	3.5 [3.0-8.3]	5.4 [3.3-9.8]	6.3 [3.0-9.6]	6.1 [3.2-12.6]	0.003
Apolipoprotein fractions					
ApoA1 (mg/dL)	142.4 ± 22.4	157.0 ± 22.9	154.2 ± 24.8	151.0 ± 24.3	< 0.001
ApoA2 (mg/dL)	32.3 ± 4.9	40.6 ± 7.0	39.6 ± 6.6	39.8 ± 6.8	< 0.001
ApoB (mg/dL)	98.0 [86.0-115.0]	89.5 [76.8-99.0]	83.0 [73.5-93.0]	79.0 [74.0-92.0]	< 0.001
ApoC2 (mg/dL)	6.0 [5.2-6.9]	4.6 [3.6-5.7]	4.3 [3.3-5.3]	4.0 [3.4-5.0]	< 0.001
ApoC3 (mg/dL)	13.8 [12.2-16.9]	9.7 [7.0-11.1]	8.3 [6.6-9.6]	7.0 [6.0-9.3]	< 0.001
ApoC3/ApoC2	2.4 [2.0-2.7]	1.9 [1.8-2.1]	1.9 [1.7-2.2]	1.9 [1.7-2.0]	< 0.001
ApoE (mg/dL)	4.7 [4.0-6.0]	3.9 [3.4-4.5]	3.6 [3.3-4.4]	3.8 [3.2-4.2]	< 0.001

Variables are expressed as number (%), means ± SD or medians [interquartile ranges].

ALP, alkaline phosphatase; ALT, alanine transaminase; Apo, apolipoprotein; AST, aspartate transaminase; BP, blood pressure; CK, creatine kinase; eGFR, estimated glomerular filtration rate; FIB-4, fibrosis-4; FLI, fatty liver index; γGT, γ-glutamyl transpeptidase; HDL-C, high-density lipoprotein cholesterol; IDL-C, intermediate density lipoprotein cholesterol; Lp(a), lipoprotein (a); LDL-C, low-density lipoprotein cholesterol; RLP-C, remnant-like particle cholesterol; sdLDL-C, small dense low-density lipoprotein cholesterol; TC, total cholesterol; TG, triglycerides; TRL-C, TG-rich lipoprotein cholesterol; VLDL-C, very low-density lipoproteins cholesterol.

### Changes in lipid-related parameters after the start of treatment with pemafibrate

**Figure 1:**
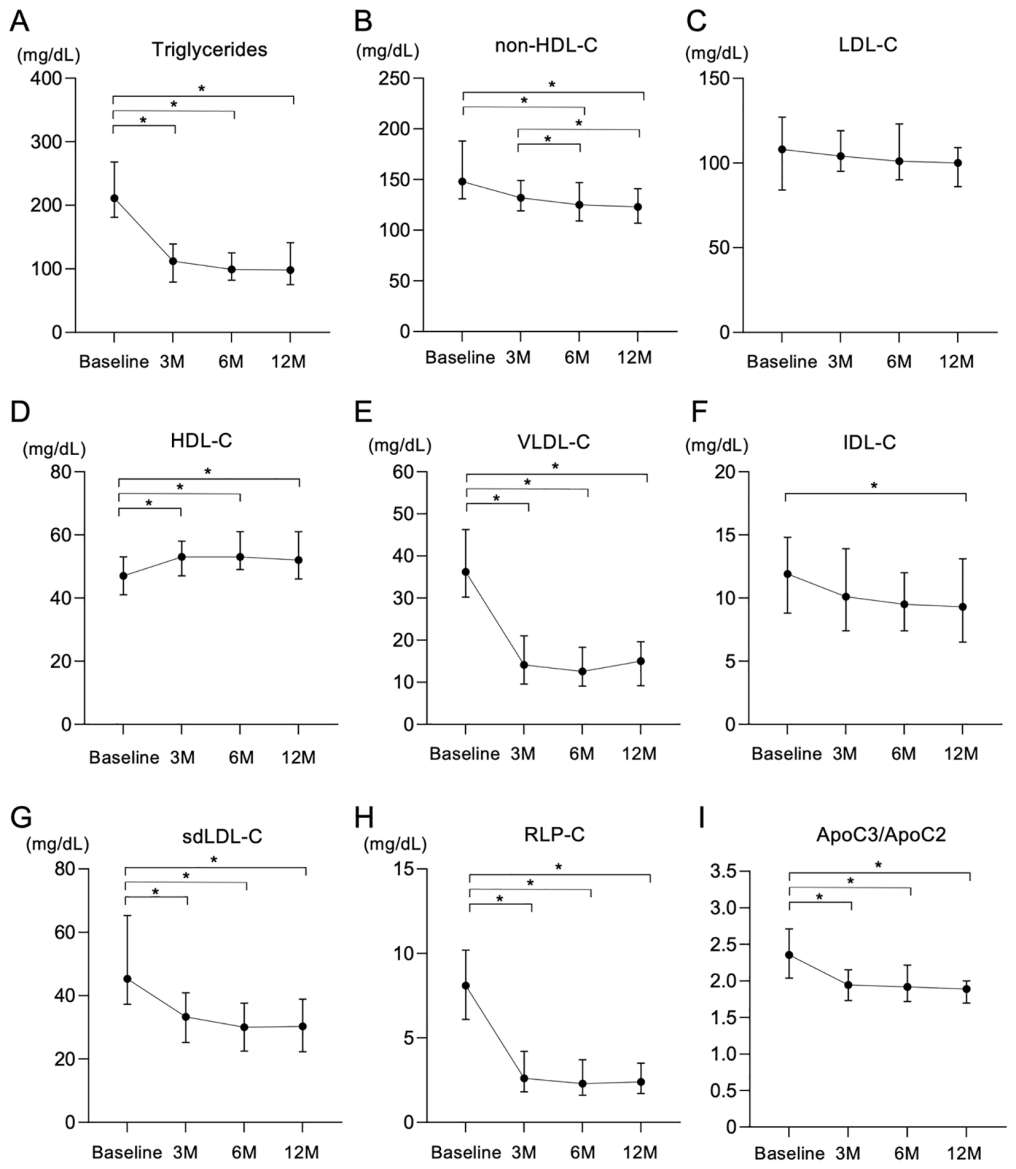
Changes in lipid-related parameters after treatment with pemafibrate for 12 months. **(A-I)** Changes in lipid-related variables including triglycerides (TG) **(A)**, non-high-density lipoprotein cholesterol (non-HDL-C) **(B)**, low-density lipoprotein cholesterol (LDL-C) **(C)**, high-density lipoprotein cholesterol (HDL-C) **(D)**, very low-density lipoprotein cholesterol (VLDL-C) **(E)**, intermediate-density lipoprotein cholesterol (IDL-C) **(F)**, small dense low-density lipoprotein cholesterol (sdLDL-C) **(G)**, remnant-like particle cholesterol (RLP-C) **(H)** and the ratio of apolipoprotein C3 to apolipoprotein C2 (ApoC3/ApoC2) **(I)** after treatment with 0.2 mg/day of pemafibrate for 12 months (M). Data are presented as medians with interquartile ranges. *p < 0.05 by the Friedman test with Dunn’s *post-hoc* test.

Treatment with pemafibrate for 3 months significantly reduced the level of TG, and the effect was maintained until 12 months after the start of treatment (**Figure 1A**). Levels of non-HDL-C were modestly, but significantly, decreased by treatment with pemafibrate for 12 months (**Figure 1B**), whereas LDL-C levels were not significantly changed over time (**Figure 1C**). Levels of HDL-C were significantly increased by treatment with pemafibrate (**Figure 1D**).

Analysis of lipoprotein fractions measured by an HPLC method showed that a significant reduction in VLDL cholesterol (VLDL-C) in the first 3 months after the start of treatment with pemafibrate remained during the whole observation period (**Figure 1E**). IDL cholesterol (IDL-C) significantly and gradually decreased during the follow-up period (**Figure 1F**).

Other atherogenic lipid parameters, including sdLDL-C (**Figure 1G**), sdLDL-C/LDL-C, RLP-C (**Figure 1H**) and TRL-C, significantly decreased after the start of treatment with pemafibrate (**Table 3**). Levels of Lp(a) significantly increased after the start of treatment with pemafibrate, though the absolute values of Lp(a) at baseline and after the start of treatment (median: 3.5-6.3 mg/dL) were relatively low [reference value: < 30 mg/dL (45–47)]. As for apolipoproteins, treatment with pemafibrate significantly increased levels of ApoA1 and ApoA2 and significantly decreased levels of ApoB, ApoC2, ApoC3 and ApoE. The ratio of ApoC3 to ApoC2 (ApoC3/ApoC2) significantly decreased after the strat of treatment with pemafibrate (**Figure 1I**).

### Changes in liver-related markers after the start of treatment with pemafibrate

**Figure 2:**
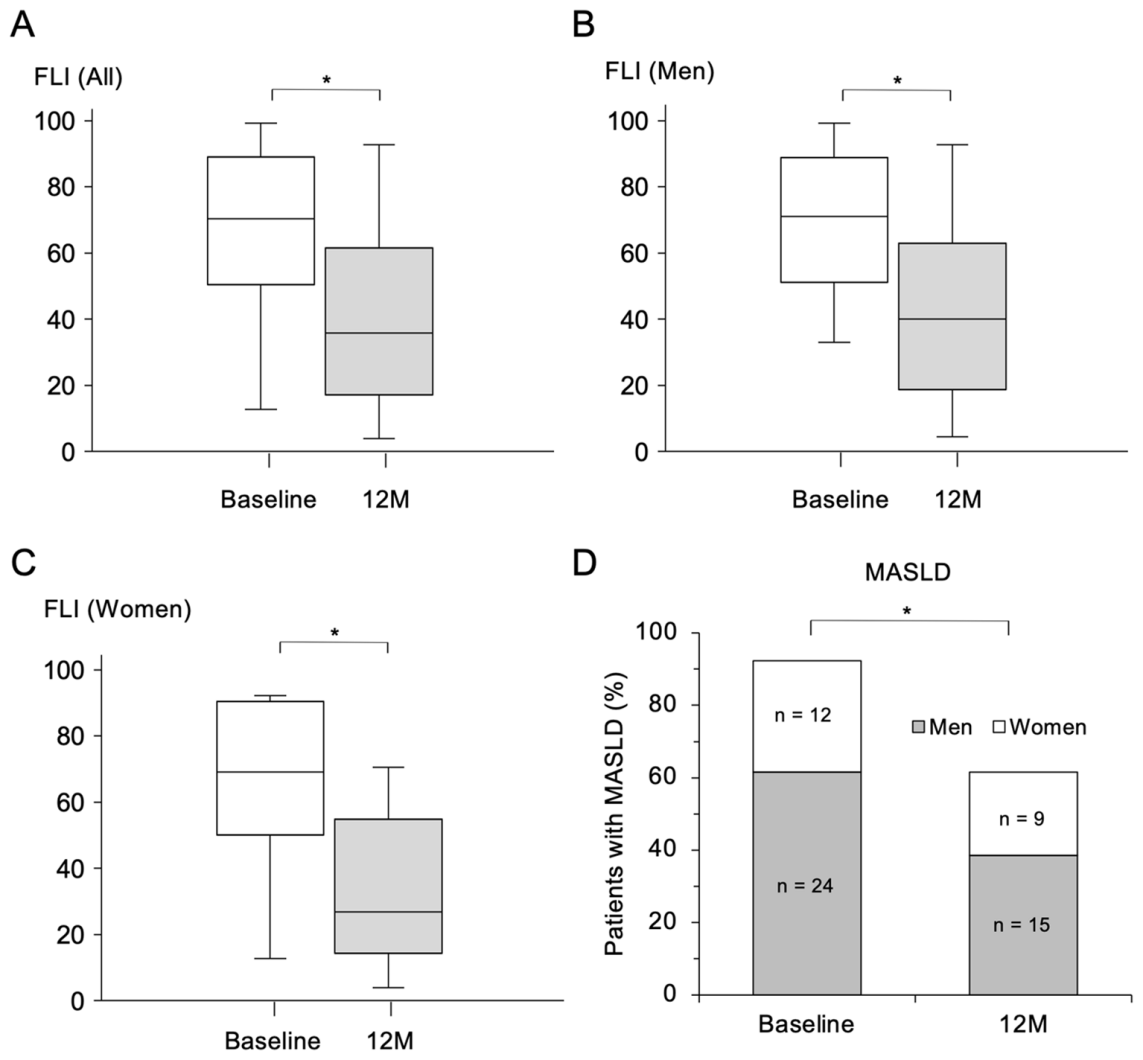
Change in FLI and prevalence of MASLD after treatment with pemafibrate for 12 months. **(A-C)** Comparisons of fatty liver index (FLI) before (at baseline) and 12 months (M) after the start of treatment with 0.2 mg/day of pemafibrate in all of the recruited patients (n = 39) **(A)** and in male patients (n = 24) **(B)** and female patients (n = 15) **(C)**. Data are presented as box-and-whisker plots. *p < 0.001 by Wilcoxon’s signed-rank test. **(D)** Comparison of the prevalence of metabolic dysfunction-associated steatotic liver disease (MASLD) before the start of treatment (at baseline) and that at 12 M after the start of treatment with pemafibrate. *p < 0.001 by McNamer’s test.

FLI, an index of hepatic steatosis, was significantly decreased by the treatment with pemafibrate for 12 months (**Figure 2A**). The improvement of FLI was observed in both men (**Figure 2B**) and women (**Figure 2C**). All of the constituent elements to calculate FLI including WC (**Supplementary Figure S1A**), BMI (**Supplementary Figure S1B**) and levels of TG (**Figure 1A**) and γGT (**Supplementary Figure S1C**) were significantly decreased by treatment with pemafibrate for 12 months. These results indicate that pemafibrate, primarily known as a TG-lowering agent, also improved all components of FLI.

There was no significant change in FIB-4 index, an index of hepatic fibrosis, after the start of treatment with pemafibrate, though the absolute value of FIB-4 index (median: 0.78-1.39) was low at baseline (reference value: < 1.3) (**Supplementary Figure S1D**). After treatment with pemafibrate for 12 months, the percentage of patients with MASLD was significantly decreased from 92.3% to 61.5% (**Figure 2D**).

## Discussion

The present study showed that treatment with pemafibrate for 12 months significantly ameliorated FLI as a marker of hepatic steatosis as well as various lipid profiles as potent atherosclerotic risk factors including levels of TG, non-HDL-C, sdLDL-C, TRL-C, RLP-C, VLDL-C and IDL-C in Japanese patients with T2DM at a clinical practice level. It has been reported that pemafibrate has beneficial effects on hepatic function with a lower frequency of hepatic impairment than other PPARα agonists (48, 49) and that pemafibrate decreases markers of liver dysfunction including ALT and γGT and markers of liver fibrosis including FIB-4 index and the AST to platelet ratio index (50–57). Treatment with pemafibrate not only reduced the levels of TG, which may contribute to atherosclerosis partly via inflammation (9), but also markedly decreased the prevalence of MASLD estimated by FLI in the present study. However, there was no significant change in FIB-4 index, presumably due to the small number of patients with advanced liver fibrosis in the present study. It has been reported that pemafibrate increases fibroblast growth factor 21 (FGF21) (58), a favorable hepatokine. FGF21 analog is currently in the clinical trial stage and has been shown to increase adiponectin, a favorable adipokine, and to ameliorate insulin resistance in humans (59). An increased circulating level of FGF21 might be involved in the results of the present study.

It has been reported that the PPARα-activating effect of pemafibrate is more than 2,500-fold stronger than that of fenofibrate, a conventionally specific PPARα agonist, and that even a dose of 0.2 mg/day of pemafibrate can affect lipid metabolism (60). Indeed, levels of TG as well as ApoB, which reflects an atherogenic lipoprotein (61), were significantly reduced over time after the start of treatment with 0.2 mg/day of pemafibrate in the present study. The findings indicate at least one of the plausible reasons why levels of sdLDL-C and RLP-C, which are highly associated with levels of TG and ApoB (62–64), were significantly and dramatically reduced by treatment with pemafibrate. Although LDL-C was not changed by treatment with pemafibrate in the present study, the sdLDL-C/LDL-C ratio was consistently decreased (**Table 3**), suggesting that pemafibrate affects the level of sdLDL-C rather than the total LDL-C level. A recent study using data from the Copenhagen General Population Study to simulate the PROMINENT trial showed that the lack of cardiovascular benefit in the original trial might be explained by a simultaneous increase in LDL-C and ApoB despite a reduction in remnant cholesterol (41). On the other hand, in the present study, there was a decrease in level of TRL-C, which is equivalent to remnant cholesterol, without accompanying an increase in level of LDL-C or ApoB (**Table 3**). Although clinical outcomes were not investigated in the present study, these changes in lipid variables may have potential effects on the prevention of atherosclerosis.

Evaluation of the effects of pemafibrate on cardiovascular outcomes was conducted in the PROMINENT trial using statin-treated patients with T2DM who had a relatively long duration of DM (≥10 years: 46.4%), mild-to-moderate hypertriglyceridemia (fasting TG ≥ 200–499 mg/dL) and HDL-C levels ≤ 40 mg/dL (22, 26). The trial was discontinued after treatment for 16 weeks (partially 48 weeks) due to a lack of sufficient reduction in cardiovascular events by treatment with 0.2 mg/day of pemafibrate compared to the treatment with a placebo in the interim analysis (22). On the other hand, in the present study, we prospectively investigated changes in lipid and metabolic factors, adverse events and indices of liver damage over time for 12 months (52 weeks) in patients with T2DM who had a relatively short duration of DM (median duration: 5.0 years), although the number of study participants was small. It is difficult to compare the results of the PROMINENT trial designed as an event-driven study with a placebo control group and the results of the present study showing a rapid and long-term improvement in FLI and atherogenic risk factors. In a subgroup analysis of the PROMINENT trial, there was a trend toward fewer primary composite endpoint in patients with duration of diabetes < 10 years than in those with duration of diabetes ≥ 10 years (22). Furthermore, the number of patients with NAFLD was also decreased by treatment with pemafibrate in the PROMINENT trial (22). It is possible that earlier intervention with pemafibrate suppresses the progression of atherosclerosis as well as MASLD.

The level of Lp(a), a potential atherogenic factor (65), increased after the start of treatment with pemafibrate in the present study. It has been speculated that PPARα agonists increase the VLDL receptor in the liver and that Lp(a) can be taken up by hepatocytes, resulting in a decrease in Lp(a) (66). It has recently been shown in a crossover study that treatment with pemafibrate for 6 months decreased Lp(a) by -17.8% (20.4 ± 30.3 to 19.1 ± 23.9 mg/dL) (67). Although the precise reason for the increase in Lp(a) in the present study is not clear, the clinical significance of a subtle increase in Lp(a) (3.5 to 6.1 mg/dL) would be small since the cutoff value of Lp(a) as an atherogenic risk factor has been reported to be 30–50 mg/dL (45–47).

It has been reported that PPARα agonists downregulate the expression of ApoC3 (68–71), a possible factor that inhibits lipoprotein lipase activity (72), and that pemafibrate also decreases ApoC3 (73), which was consistent with the results of the present study. ApoC2, which promotes the function of lipoprotein lipase, was also decreased after the start of treatment with pemafibrate in the present study. However, the ApoC3/ApoC2 ratio was reduced by treatment with pemafibrate (**Figure 1I**) as previously reported (73, 74), possibly leading to an increase in lipoprotein lipase activity and a decrease in the level of TG. Further studies are needed to determine whether pemafibrate affects the regulation of both ApoC2 and ApoC3 and, if so, how pemafibrate affects the regulation.

Treatment with pemafibrate slightly, but significantly, decreased renal function in the present study, as was also observed in the PROMINENT trial (22). The change in renal function has been reported to be reversible and recovered by discontinuation of treatment with pemafibrate (75). The level of creatine kinase was not increased over time by treatment with pemafibrate, and rhabdomyolysis was not confirmed in the present study. In addition, during the follow-up period, there was no significant change in HbA1c, but there was a slight decrease in fasting glucose. These findings are consistent with the results of a previous study showing that pemafibrate is selective for PPARα with less adverse effects than those of conventional fibrates (67).

It is noteworthy that pulse rate in the enrolled patients was significantly decreased by treatment with pemafibrate (**Table 3**). It has been shown that an increase in resting pulse rate (or heart rate) is associated with autonomic dysfunction in patients with T2DM (76). While the effects of pemafibrate on reducing pulse rate have not been established yet, the finding in the present study suggests that pemafibrate has the potential to improve autonomic nervous dysfunction independently of glycemic management in patients with T2DM. Further studies are warranted to investigate the causal relationship and underlying mechanisms between pemafibrate treatment and cardiac autonomic dysfunction in patients with T2DM.

There are several limitations in the present study. First, the sample size was small, and no placebo group was included because the present study was designed as a single-center prospective and real-world study conducted at a clinical practice level. Second, detection of SLD estimated by FLI as a surrogate marker, but not diagnosis by invasive liver biopsy samples and additional image modalities, was used in the present study. Third, since only Japanese patients were enrolled, the results obtained in the present study might not be applicable to other races. Fourth, although this study was conducted in patients with T2DM and hypertriglyceridemia as a high-risk group for ASCVD, the impact of pemafibrate should be evaluated in several conditions including less glycemic management and severe liver damage. Finally, while this study could not address the underlying mechanisms by which pemafibrate improves MASLD and atherogenic lipid profiles, its potential involvement in interorgan crosstalk as proposed in the emerging concepts of cardiovascular-kidney-metabolic syndrome (77) and the liver-spleen axis (78) warrants further investigation.

In conclusion, pemafibrate ameliorates MASLD estimated by FLI in addition to various atherogenic lipoid profiles in Japanese hypertriglyceridemia patients with T2DM. An early intervention with pemafibrate might contribute to prevention of the development of MASLD and ASCVD.

## Data Availability

The original contributions presented in the study are included in the article/**Supplementary Material**. Further inquiries can be directed to the corresponding author.
