# Supplementary material for: Treatment with pemafibrate ameliorates fatty liver index and atherogenic lipid profiles in Japanese patients with type 2 diabetes mellitus
Source: Front Endocrinol (Lausanne). 2025 Jul 17;16:1496671. doi: 10.3389/fendo.2025.1496671 (PMC12310470; doi:10.3389/fendo.2025.1496671)
Supplement: Supplementary file 1 [file DataSheet1.pdf]

Supplementary Figure S1

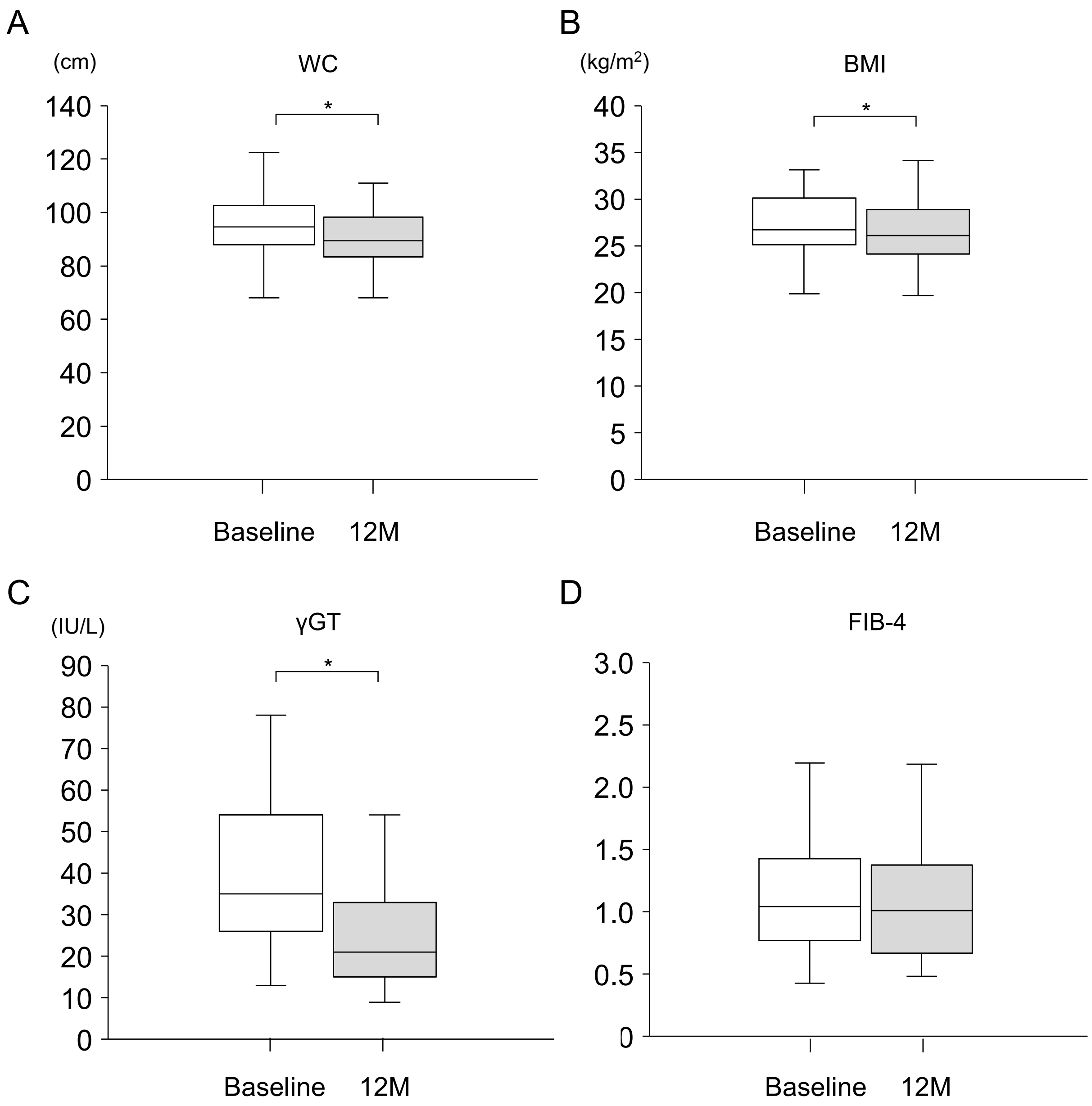

Supplementary Figure S1. Changes in constituent elements of FLI or FIB-4 after treatment with pemafibrate.

A-C. Changes in constituent elements to calculate FLI, including waist circumference (WC) (A), body mass index (BMI) (B) and serum levels of γ-glutamyl transpeptidase (γGT) (C) before (baseline) and after treatment with 0.2 mg/day pemafibrate for 12 months (M). **D.** Change in fibrosis-4 (FIB-4), an index of hepatic fibrosis, before (baseline) and after the treatment with 0.2 mg/day pemafibrate. Data are presented as box-and-whisker plots. *p < 0.001 by Wilcoxon single rank test.
